# Acceptability and HIV Prevention Benefits of a Peer-Based Model of Rapid Point of Care HIV Testing for Australian Gay, Bisexual and Other Men Who Have Sex with Men

**DOI:** 10.1007/s10461-017-1888-1

**Published:** 2017-08-28

**Authors:** David Leitinger, Kathleen E. Ryan, Graham Brown, Alisa Pedrana, Anna L. Wilkinson, Claire Ryan, Margaret Hellard, Mark Stoové

**Affiliations:** 1 Centre for Population Health, Burnet Institute, 85 Commercial Road, Melbourne, VIC 3004 Australia; 2 Victorian AIDS Council, South Yarra, VIC Australia; 3 School of Public Health and Preventive Medicine, Monash University, Melbourne, VIC Australia; 4 Australian Research Centre in Sex, Health and Society, LaTrobe University, Melbourne, VIC Australia; 5 Department of Nutrition, Harvard T.H. Chan School of Public Health, Cambridge, MA USA; 6 Alfred Health Infectious Disease Department, Alfred Hospital, Melbourne, VIC Australia

**Keywords:** HIV, Rapid testing, MSM, Peer, Australia

## Abstract

Frequent HIV testing among gay, bisexual and other men who have sex with men (GBM) is a strategic priority for HIV prevention in Australia. To overcome barriers to testing in conventional clinical services, Australia recently introduced peer HIV rapid point of care (RPOC) testing services for GBM. This mixed methods evaluation describes client acceptability and HIV prevention benefits of a peer HIV testing model. Most aspects of the service model were overwhelmingly acceptable to clients. Two-thirds of survey participants reported preferring testing with peers rather than doctors or nurses and over half reported learning something new about reducing HIV risk. Focus group findings suggested peer-delivered HIV RPOC testing reduced stigma-related barriers to frequent testing and provided novel opportunities for GBM to openly discuss HIV prevention and sexual practices, enhancing their HIV risk-reduction knowledge. Analysis of survey data suggested knowledge transfer occurred particularly among younger and less gay community-attached GBM.

## Resumen

La prueba frecuente de detección del VIH entre los hombres gay, bisexuales y hombres que tienen sexo con hombres (GBH) es una prioridad estratégica para la prevención del VIH en Australia. Para superar barreras a esta prueba de detección en servicios clínicos convencionales, Australia recientemente introdujo servicios rápidos de prueba de detección del VIH atendidos por pares (iguales) de los GBH. Esta evaluación de métodos mixtos describe la aceptación del cliente y los beneficios de la prevención del VIH de un modelo de pruebas de detección del VIH realizadas por pares. La mayoría de los aspectos de este modelo de servicio están rotundamente aceptados por los clientes. Dos tercios de los participantes en el estudio indicaron su preferencia por pruebas realizadas por pares en vez de médicos o enfermeras, y más de la mitad comunicaron haber aprendido algo nuevo sobre la reducción del riesgo de contraer el VIH. Los resultados de grupos de enfoque sugieren que servicios rápidos de las pruebas de detección del VIH realizadas por pares reducen barreras relacionadas con el estigma del VIH y dan nuevas oportunidades a los GBH discutir abiertamente la prevención del VIH y prácticas sexuales, mejorando su conocimiento de reducción de riesgos del VIH. El análisis de los datos del estudio sugiere que la mejoría de conocimiento ocurre particularmente entre los GBH mas jóvenes y menos conectados a la comunidad gay.

## Introduction

Like much of the developed world [1], Australia has seen a resurgent HIV epidemic among gay, bisexual and other men who have sex with men (GBM). While annual HIV diagnoses have stabilized over recent years [2], this represents a 43% increase in annual diagnoses compared to 1999 [3]. The Australian epidemic among GBM is disproportionately driven by the estimated 9–31% of HIV infections among GBM that remain undiagnosed [4–6], which may be responsible for at least half the new infections among GBM in Australia each year [7]. In keeping with the UNAIDS 90-90-90 target to end the global HIV/AIDS epidemic by 2030 [8], Australia has placed a strategic emphasis on frequent HIV testing among GBM as the foundation of a strengthened cascade of care [9]. Early diagnosis through more frequent testing will reduce the prevalence of undiagnosed HIV, as well as facilitating timely antiretroviral therapy commencement, subsequently reducing the likelihood of onward transmission [10, 11] and improving individual health outcomes [12].

HIV testing recommendations in many countries are based on individual sexual and other risk [13–16]. In Australia, guidelines recommend quarterly HIV testing for all ‘high-risk’ GBM, who are those reporting any condomless anal intercourse (CAI), anal sex with more than 10 men, group sex or drug use during sex in the last six months [17]. Nevertheless, recent data from local clinics with high HIV caseloads show that only half of all GBM returned for testing within 12 months [18], while 15 and 36% of high-risk GBM returned within three and six months, respectively [19]. Although HIV testing in Australia is predominantly free or low cost through government healthcare subsidies, the almost exclusive delivery of HIV testing through laboratory-based serology in primary care clinical settings raises barriers to frequent testing [19, 20]. For Australian GBM, barriers include the inconvenience and anxiety caused by waiting and returning for serology results at subsequent appointments, difficulties finding clinic appointments and discomfort discussing sexual history with doctors [21, 22].

Alternative testing models have helped overcome barriers to testing for GBM internationally [23]. HIV rapid point of care (RPOC) testing has advantages over conventional testing. These include the convenience of receiving test results in the same appointment and the potential for their delivery in non-clinical environments. As a result, HIV RPOC testing and has been shown to increase testing uptake among GBM [24]. When trialed in conventional sexual health centers, HIV RPOC testing was highly acceptable to Australian GBM, but did not produce sustained improvements in testing frequency [25–27]. The limited impact on testing frequency may have related to largely unchanged clinical service delivery models, which did not optimize the convenience of HIV RPOC testing or address psychosocial barriers associated with these settings [26]. These findings suggest alternative approaches to HIV RPOC testing are warranted. Peer-delivered conventional sexual health services have reduced psychosocial barriers to testing for Australian GBM [28], and peer-led education and referral programs have long been used to increase testing uptake internationally [29]. Little is known, however, about the acceptability and impact of peer-delivered HIV RPOC testing for GBM.

In 2012, Australia’s Therapeutic Goods Administration (TGA) approved the first HIV RPOC test device [30], allowing HIV RPOC testing to be offered in entirely peer-delivered services for the first time. In August 2013, PRONTO! opened as Victoria’s only peer-delivered HIV RPOC testing service with the primary aim of supporting GBM to test for HIV more frequently. This paper uses mixed methods data from clients attending PRONTO! during its first 14 months of operation to describe the acceptability of the peer model and explore its contribution to HIV prevention, aiming to guide PRONTO!’s ongoing refinement and the expansion of HIV RPOC testing in Australia.

## Methods

### Setting

PRONTO! is located in an inner suburb of Melbourne in proximity to several gay social and sex-on-premises venues. PRONTO! opened on August 15th 2013 as a 2-year trial (August 2013-August 2015) funded by the Victorian Department of Health and Human Services and operated by the Victorian AIDS Council.

### The PRONTO! HIV RPOC Testing Model

The peer-delivered HIV RPOC testing model at PRONTO! has been described previously [31]. Briefly, PRONTO! provides free HIV RPOC testing (Trinity Biotech Uni-Gold HIV 1/2) according to manufacturer’s instructions [32]. The service operates on Saturday (10:30 am–2:30 pm) and weekday evenings (4:00–8:00 pm) and is staffed by one receptionist and two test facilitators. Almost all staff members identify as GBM and the service is advertised as being run by and for gay men. Peer testers do not necessarily have clinical qualifications but are trained in HIV pre- and post-test discussion and test device conduct.

Appointments are 30 min in duration, beginning with a self-completed behavioral questionnaire (described below) before consultation with a test facilitator for the HIV RPOC test. Following a discussion about the HIV testing device and possible results, a finger-prick blood specimen is collected and the HIV RPOC test started. During the 10-min test incubation time, the test facilitator engages clients in discussions about recent HIV/STI risk exposures, HIV risk reduction strategies and other information or referrals guided by clients’ needs.

All clients receiving non-reactive test results are encouraged to re-test quarterly. Following a reactive test result, venipuncture is performed for confirmatory laboratory-based serology and results provided one week later. Clients receiving a confirmed positive diagnosis are referred to specialist services of their choice for follow up care. If an HIV RPOC test result is invalid (neither non-reactive nor reactive) then it is repeated.

During the period of data collection, only HIV (and for a short period, syphilis) RPOC testing was offered at PRONTO!. Since February 2016, PRONTO! has integrated parallel HIV serology, full sexually transmissible infection (STI) screening (syphilis serology and throat swab, anal swab and first pass urine PCR for *Chlamydia trachomatis* and *Neisseria gonorrhoeae*), HIV pre-exposure prophylaxis (PrEP) initiation and maintenance and shares its premises with a trans and gender diverse health service.

### Quantitative Data Collection and Analysis

Two methods of quantitative data collection were used to evaluate trial outcomes. First, a sentinel surveillance system individually linked client test records, test outcomes and brief behavioral surveys completed at each testing event. Second, two evaluation surveys were completed by PRONTO! clients; the first commencing three months after the service was established (round one: 12/11/2013–31/01/2014) and the second commencing six months later (round two: 15/05/2014–06/10/2014). The period of data analysis in this paper is from the date PRONTO! opened (15/08/2013) to the end of the second evaluation survey (06/10/2014).

#### Sentinel Surveillance

The Victorian Primary Care Network for Sentinel Surveillance on Blood Borne Viruses and Sexually Transmissible Infections (VPCNSS) was established in 2006 to monitor HIV testing frequency, test outcomes and risk behaviors from people testing for HIV (see system attributes detailed elsewhere [33]). PRONTO! was integrated into VPCNSS at the time of its establishment. All clients attending PRONTO! for HIV RPOC testing self-completed a 23-item behavioral questionnaire at reception asking questions on demographics, testing history and sexual behaviors. Behavioral data were matched to their test record and prospectively linked using a unique client identifier.

Descriptive analyses characterized sexually active GBM (those reporting any male sex partners over the past six months) receiving HIV RPOC testing at PRONTO! during the study period. If a client tested more than once during this period, only their first record was included in this analysis.

#### Evaluation Survey

GBM receiving an HIV RPOC test were invited to self-complete a de-identified online survey about their experience at PRONTO!. Survey items (85 in round one and 73 in round two) included questions about barriers to HIV testing experienced before first attending PRONTO! and asked participants to respond to statements on a five-point scale ranging from strongly agree to strongly disagree about the acceptability of features of the service model—including its accessibility, physical environment, testing processes and peer staffing—and perceived improvement in participants’ HIV risk reduction knowledge. Participants completing a survey in round one were not reimbursed, while those in round two were offered a $20 gift voucher to encourage participation.

Analyses were restricted to males aged 18 years and over reporting any sex with males in the past six months or self-identifying as gay/bisexual. Survey respondent demographics, HIV testing history and sexual behavior were compared with sentinel surveillance data to assess the representativeness of evaluation survey participants using a two-sample test of proportions. Descriptive analysis of participant demographics, testing history, sexual risk, acceptability outcomes and HIV risk reduction knowledge was performed, pooling data from both survey rounds. Multivariate logistic regression examined correlates of two key anticipated benefits of the peer-led model: (1) preferring to test with peers over sexual health doctors or nurses as a measure of the peer model’s acceptability; and (2) improved HIV risk reduction knowledge as a potential HIV prevention benefit, as observed in other peer-delivered programs [34, 35]. The regression model was adjusted for selected key covariates (age, country of birth, proportion of friends who are gay men, previous HIV testing, number of anal sex partners, condom use with regular and casual partners, group sex), consistent with behavioral surveillance in Victoria [33] and previously identified as correlates of HIV testing uptake among Australian GBM [36], as well as the number of tests at PRONTO!. The model was validated with the Pearson goodness of fit test.

All analyses were performed using Stata 13 (StataCorp LP, College Station, Texas, USA) with statistical significance set at p < 0.05.

### Qualitative Data Collection and Analysis

#### Focus Group Discussions with Clients

GBM completing evaluation surveys were invited to participate in focus groups. Two focus group discussions of approximately 90 min in length and with between five and 10 participants occurred after each survey round. Focus group schedules explored a range of themes that included the acceptability, benefits and limitations of the PRONTO! service model. Discussions were recorded and transcribed verbatim, with participants given pseudonyms. At the time of invitation, participants were informed they would be reimbursed $40 for their time and travel expenses. Because the evaluation surveys were de-identified, focus group participants could not be linked to their survey responses.

Transcripts were analyzed with NVivo 11 (QSR International Pty Ltd, Doncaster, Australia) using a conventional thematic analysis framework. While the focus group discussions ranged broadly, analysis for this study was restricted to discussions of the peer model. Two researchers independently coded the transcripts, continuously revising codes before grouping them into emergent themes relating to the peer model. Participant feedback on findings was not sought.

### Ethical Statement

The Alfred Health Human Ethics Committee (No. 297/13) granted ethical approval for the evaluation survey and focus group discussions, and the Victorian Government Department of Human Services Human Research Ethics Committee (No. 52/05) approved sentinel surveillance.

## Results

### Sentinel Surveillance

Between 15/08/2013 and 06/10/2014, 1355 GBM attended PRONTO and received 1738 HIV RPOC tests. Two hundred and ninety-three (20.7%) clients tested more than once (analysis of return testing at PRONTO! has been described previously [31]) and 14 (1.0%) received a reactive HIV RPOC test result. The median age of GBM testing at PRONTO! was 30.5 years, more than half were born in Australia and one in 25 identified as Aboriginal or Torres Strait Islander. Over 80% of participants reported having ever had an HIV test prior to attending PRONTO! and approximately three-quarters of these reported testing within the previous 12 months. Sexual histories consistent with high-risk classification were common [17]; in the past six months, approximately one in 10 GBM reported more than ten anal sex partners, over 90% reported casual male sex partners and nearly half reported CAI with casual male sex partners. One-third reported group sex and one in seven reported recreational drug use during sex in the previous six months (Table 1).

**Table 1:** Comparison of demographic, testing history and sexual risk data between sentinel surveillance and evaluation surveys one and two combined

	Sentinel surveillance^a^	Evaluation surveys^b^	Two-sample test of proportions	
	n (%)	n (%)	z score	p value
Total included participants	1355 (100)	416 (100)	–	–
Number receiving a reactive HIV RPOCT result	14 (1.0)	–	–	–
Age				
Median (interquartile range)	30.5 (26–38)	30 (26–39)	–	–
≤29 years^c^	644 (47.5)	187 (45.0)	0.89	0.37
30–39 years	431 (31.8)	134 (32.2)	−0.15	0.88
40–49 years	193 (14.2)	66 (15.9)	−0.86	0.39
50+ years	87 (6.4)	29 (7.0)	−0.43	0.67
Born in Australia	776 (60.3)	270 (64.9)	−1.68	0.09
Identify as aboriginal and/or Torres Strait Islander	52 (3.9)	2 (0.5)	**3.50**	**<0.01**
Ever tested for HIV before first test at PRONTO! (self-reported)	1121 (83.1)	352 (84.8)	−0.82	0.42
Tested for HIV within the 12 months before first testing at PRONTO!, if ever tested	732 (75.9)	266 (72.8)	1.16	0.25
Number of anal sex partners in the last 6 months				
None	73 (5.4)	22 (5.3)	0.08	0.94
One man	255 (18.9)	63 (15.1)	1.76	0.08
2–10 men	769 (64.4)	250 (60.1)	1.59	0.11
11+ men	152 (11.3)	81 (19.5)	−**4.32**	**<0.01**
Any regular male sex partners (RMPs) in the last 6 months	905 (73.8)	282 (71.2)	1.02	0.31
Any condomless anal intercourse with RMPs^d^	519 (64.6)	180 (63.8)	0.24	0.81
Any casual male sex partners (CMPs) in the last 6 months	1239 (93.0)	366 (92.9)	0.07	0.95
Any condomless anal intercourse with CMPs^e^	509 (45.3)	163 (44.5)	0.27	0.79
Any group sex in the last 6 months	452 (33.5)	174 (42.4)	−**3.30**	**<0.01**
Any drug use during sex in the last 6 months	172 (14.9)	75 (18.4)	−1.67	0.10
Ever injected a drug	46 (3.4)	17 (4.1)	−0.67	0.50
At least half of friends are gay men	–	304 (73.1)	–	–
Reported experiencing barriers to HIV testing before first testing at PRONTO!	–	138 (33.3)	–	–

Bold values indicate statistical significance (p < 0.05)

^a^Data for the period 15/08/2013–06/10/2014

^b^Data for the period 12/11/2013–31/01/2014 (survey one) and 15/05/2014–06/10/2014 (survey two) combined

^c^Minimum age for sentinel surveillance and the evaluation survey is 16 and 18 years, respectively

^d^If reported any RMPs in the last 6 months

^e^If reported any CMPs in the last 6 months

### Evaluation Survey

During the recruitment periods for survey rounds one and two, 263 and 770 unique eligible GBM clients accessed HIV RPOC testing at PRONTO!, respectively. Of these, 118 (44.9%) in round one and 298 (38.7%) in round two completed a survey and were included in this analysis. Evaluation survey participants were similar in demographics, testing history and sexual risk profile to the GBM accessing HIV RPOC testing at PRONTO! as reported in sentinel surveillance data with the exception that significantly fewer identified as Aboriginal or Torres Strait Islander (z = 3.50, p < 0.01) or reported more than 10 anal sex partners (z = −4.32, p < 0.01), while significantly more reported group sex (z = −3.30, p < 0.01). In addition over half of evaluation survey respondents reported at least half of their friends to be gay men and one-third reported experiencing barriers to HIV testing prior to first attending PRONTO! (questions not asked in sentinel surveillance questionnaires) (Table 1).

#### Acceptability of the Peer Based Model and its Contribution to Prevention

Service acceptability was overwhelmingly high across nearly all outcomes examined in the evaluation survey. More than 90% of participants agreed or strongly agreed that they were comfortable waiting for their result in the consultation room with peer test facilitators, and that peer test facilitators were competent, professional and able to provide the information and referrals participants required. Nearly all indicated they would be likely to return to PRONTO! for HIV testing and four fifths indicated that they would choose PRONTO! in preference to other HIV testing services (Table 2). Two-thirds of the participants indicated that they preferred testing with peer test facilitators compared to sexual health doctors or nurses and more than half reported having a better understanding of HIV risk reduction after testing at PRONTO!.

**Table 2:** Evaluation survey service acceptability and knowledge acquisition outcomes from surveys one and two combined

	Strongly agree	Agree	Neither agree nor disagree	Disagree	Strongly disagree
	n (%)	n (%)	n (%)	n (%)	n (%)
I was comfortable waiting in the consultation room with the test facilitator to receive my test result	303 (72.8)	100 (24.0)	8 (1.9)	3 (0.7)	2 (0.5)
[The test facilitators] delivered the test and pre- and post-test counseling competently	284 (68.3)	124 (29.8)	6 (1.4)	1 (0.2)	1 (0.2)
[The test facilitators] managed the whole testing experience professionally	312 (75.0)	100 (24.0)	2 (0.5)	0 (0.0)	2 (0.5)
[The test facilitators] were able to answer my questions or provided referrals to other sources of help or information	234 (56.3)	147 (35.3)	31 (7.5)	2 (0.5)	2 (0.5)
I am likely to return to PRONTO! for HIV testing	332 (80.0)	61 (14.7)	14 (3.4)	4 (1.0)	4 (1.0)
Overall, after having tested at PRONTO!, I will choose PRONTO! in preference to other HIV testing services in the future	238 (57.5)	109 (26.3)	55 (13.3)	10 (2.4)	2 (0.5)
I have a better understanding of/learnt something new about HIV risk and ways to reduce my risk of HIV infection	108 (26.0)	108 (26.0)	159 (38.2)	31 (7.5)	10 (2.4)
Overall, I prefer testing with a peer test facilitator rather than a sexual health doctor or nurse	179 (43.1)	92 (22.2)	128 (30.8)	10 (2.4)	6 (1.4)

In adjusted multivariate analysis, only reporting group sex within the last six months was associated with participants’ being less likely to prefer HIV testing with peers to sexual health doctors or nurses (aOR 0.42, 95% CI 0.24–0.71). Being aged 18–29 years (compared with 40+ years) (aOR 2.24, 95% CI 1.28–3.90), reporting testing at PRONTO! more than once (aOR 1.71, 95% CI 1.03–2.85) and reporting less than half of their friends to be gay men (aOR 1.66, 95% CI 1.06–2.60) were associated with improved HIV risk and risk-reduction knowledge (Table 3).

**Table 3:** Adjusted logistic regression analysis of correlates of preferring peer test facilitators or HIV risk and risk reduction knowledge acquisition among evaluation survey participants (surveys one and two combined)

	Overall, I prefer testing with a peer test facilitator rather than a sexual health doctor or nurse				I have a better understanding of/learnt something new about HIV risk and ways to reduce my risk of HIV infection			
	Neutral/disagree^a^	Agree^b^			Neutral/disagree^a^	Agree^b^		
	n (%)	n (%)	OR (95% CI)	aOR^c^ (95% CI)	n (%)	n (%)	OR (95% CI)	aOR^c^ (95% CI)
Age								
18–29 years	46 (35.9)	120 (49.8)	**1.80 (1.03**–**3.10)**	1.60 (0.88–2.92)	63 (34.6)	103 (55.1)	**2.50 (1.46**–**4.27)**	**2.24 (1.28**–**3.90)**
30–39 years	47 (36.7)	70 (29.0)	1.02 (0.58–1.80)	1.01 (0.55–1.89)	67 (36.8)	50 (26.7)	1.14 (0.65–2.01)	1.26 (0.70–2.28)
40+ years	35 (27.3)	51 (21.2)	Ref	Ref	52 (28.6)	34 (18.2)	ref	ref
Country of birth								
Australia	88 (68.8)	154 (63.9)	0.80 (0.51–1.27)	0.93 (0.56–1.54)	117 (64.3)	62 (33.2)	1.12 (0.73–1.72)	1.25 (0.79–1.99)
Other	40 (31.2)	87 (36.1)	Ref	Ref	65 (35.7)	125 (66.8)	Ref	Ref
Proportion of friends who are gay men								
Less than half	51 (39.8)	121 (50.2)	1.52 (0.99–2.35)	1.43 (0.90–2.29)	71 (39.0)	101 (54.0)	**1.84 (1.21**–**2.78)**	**1.66 (1.06**–**2.60)**
Half or more	77 (60.2)	120 (49.8)	Ref	Ref	111 (61.0)	86 (46.0)	Ref	Ref
HIV testing history								
Never tested	14 (10.9)	24 (10.0)	Ref	Ref	10 (5.5)	28 (15.0)	Ref	Ref
Within the past 12 months	91 (71.1)	158 (65.6)	1.01 (0.50–2.06)	1.38 (0.61–3.09)	133 (73.1)	116 (62.0)	**0.31 (0.14**–**0.67)**	0.43 (0.18–1.02)
More than 12 months ago	23 (18.0)	59 (24.5)	1.50 (0.66–3.39)	1.89 (0.72–4.98)	39 (21.4)	43 (23.0)	**0.39 (0.17**–**0.92)**	0.61 (0.23–1.60)
Ever experienced barriers to HIV testing								
No	91 (70.1)	154 (63.9)	Ref	Ref	122 (67.0)	123 (65.8)	Ref	Ref
Yes	37 (28.9)	87 (36.1)	1.39 (0.87–2.21)	1.34 (0.80–2.23)	60 (33.0)	64 (34.2)	1.06 (0.69–1.63)	1.10 (0.68–1.79)
Number of tests at PRONTO!								
One only	96 (75.0)	182 (75.5)	Ref	Ref	144 (79.1)	134 (71.7)	Ref	Ref
More than one	32 (25.0)	59 (24.5)	0.97 (0.59–1.60)	1.05 (0.63–1.78)	38 (20.9)	53 (28.3)	1.50 (0.93–2.42)	**1.71 (1.03**–**2.85)**
Number of anal sex partners^d^								
One man	20 (15.6)	40 (16.6)	Ref	Ref	30 (16.5)	30 (16.0)	Ref	Ref
2–10 men	82 (64.1)	150 (62.2)	0.91 (0.50–1.67)	1.19 (0.55–2.59)	111 (61.0)	121 (64.7)	1.09 (0.62–1.92)	1.75 (0.84–3.64)
11+ men	26 (20.3)	51 (21.2)	0.98 (0.48–2.01)	1.90 (0.72–4.94)	41 (22.5)	36 (19.3)	0.88 (0.45–1.73)	1.63 (0.67–3.97)
Condom use: anal sex, regular partners								
Consistent condom use	30 (23.4)	68 (28.5)	Ref	Ref	43 (23.8)	55 (29.6)	Ref	Ref
Inconsistent condom use	62 (48.4)	101 (42.3)	0.72 (0.42–1.23)	0.68 (0.37–1.24)	84 (46.4)	79 (42.5)	0.74 (0.44–1.22)	0.88 (0.50–1.55)
No anal sex with regular partners	36 (28.1)	70 (29.3)	0.86 (0.48–1.55)	0.71 (0.36–1.38)	54 (29.8)	52 (28.0)	0.75 (0.43–1.31)	0.78 (0.42–1.43)
Condom use: anal sex, casual partners								
Consistent condom use	73 (57.0)	119 (49.4)	Ref	Ref	93 (51.1)	99 (52.9)	Ref	Ref
Inconsistent condom use	49 (38.3)	103 (42.7)	1.29 (0.82–2.02)	1.38 (0.81–2.33)	78 (42.9)	74 (39.6)	0.89 (0.58–1.37)	0.93 (0.57–1.52)
No anal sex with casual partners	6 (4.7)	19 (7.9)	1.94 (0.74–5.10)	1.50 (0.46–4.86)	11 (6.0)	14 (7.5)	1.20 (0.52–2.77)	1.22 (0.42–3.58)
Group sex								
None	56 (43.8)	150 (62.2)	Ref	Ref	91 (50.0)	115 (61.5)	Ref	Ref
Any	72 (56.2)	91 (37.8)	**0.47 (0.31**–**0.73)**	**0.41 (0.24**–**0.70)**	91 (50.0)	72 (38.5)	**0.63 (0.41**–**0.95)**	0.69 (0.40–1.10)

Bold values indicate statistical significance (p < 0.05)

^a^Includes the categories “neither agree nor disagree”, “disagree” and “strongly disagree”

^b^Includes the categories “agree” and “strongly agree”

^c^Adjusted for all variables

^d^Clients not reporting any anal sex partners excluded due to collinearity with condom use variable

### Client Focus Groups

Of the 416 PRONTO! clients completing the evaluation surveys, 236 (56.7%) consented to be contacted about participating in focus group discussions and were sent an email inviting them to participate in the focus groups. Twenty-six of these attended one of four focus group discussions; 10 following survey round one and 16 following survey round two. The median age of focus group participants was 29 years (range 20–46 years). Eleven reported testing at PRONTO! once only, six had tested two times and six had tested three times (three did not report how many times they had tested at PRONTO!).

#### A Gay Man’s Testing Service

Although half of the participants were not aware at their first visit that PRONTO! was a peer-led service, participants were near unanimous in reporting a sense of comfort and safety from the knowledge that PRONTO! was operated for gay men:

being a service that’s just for gay men, I feel to me that’s enough I guess. ‘Cause, if they’re going to be working with gay men they’re going to be, they’ll probably be OK with it

– Gus, round two, 25 years

A minority of participants indicated that knowing that PRONTO! was a service tailored to gay men was enough to make them comfortable there. For these men staff members need not have been gay male peers, provided they were not judgmental and had a good understanding of gay sex and sexual health. More commonly, however, participants explicitly stated they preferred a peer-led service. These participants expressed the view that peers were more relatable and better able to understand the practices and concerns of other gay men, which helped them feel comfortable disclosing sensitive sexual history information:

I think there’s that experiential understanding that another gay man brings to it that makes it easier to relate to him and to perhaps be completely honest about what it is that you do as well, so, because it’s not, you know, you can have a theoretical understanding but an experiential one can’t be beat

– James, round two, 26 years

#### The Client-Provider Relationship

Participants highly valued the perceived non-judgmental and non-prescriptive attitude of PRONTO! staff during pre- and post-test discussion about sexual practices. Participants contrasted their experience at PRONTO! with prior tests at conventional clinical services, where they described feeling judged when discussing their sexual history and felt a greater power differential between them and their test provider:

it’s a bit like teacher-student in [a clinical] setting but with, at PRONTO!, it was almost like my mate for like half an hour

– Hugh, round one, 24 years

Other than the perception of a more even client-provider power balance, participants described the client-provider relationship during the pre- and post-test conversation at PRONTO! as less formal compared to experiences at other testing services. This relaxed style was generally preferred because the more varied topics covered (not always related to sexual health or the HIV test) helped clients relax and reduced the anxiety of waiting for the test result. For some participants this created a sense of a more personalized service:

the fact that it’s peer-led, it’s people that aren’t seeing you just as a patient or a client, there’s a kind of more of an equal treatment, and I found they were more able to just relate to you in a very casual manner, so I really liked that.’Cause you know, sometimes you go into a clinic and it might be a nurse treating you or testing you and you kind of feel like a number

– James, round two, 26 years

In contrast, one participant stated he preferred more formal relationships with doctors and nurses. For this participant, formality gave a sense of objectivity on the part of the test provider, which could make it easier to disclose personal information.

#### A Greater Opportunity for Discussion

Other than a greater sense of comfort disclosing their sexual history, a prominent theme through all focus groups was that PRONTO! provided an opportunity for more in-depth and potentially informative pre- and post-test conversations compared to their experiences at other testing services such as a general practitioner (GP). This was in part attributed to the comparatively long duration of test consultations (30 min). In addition to the time for discussion afforded by the HIV RPOC test incubation period, several participants remarked that, unlike conventional testing consultations, the incubation period helped focus attention on HIV risk and provided an opportunity to engage in a detailed conversation about HIV and sexual health:

the fact that you’ve got that wait time where you’re having conversation with them waiting for the test results, you certainly don’t do that with a GP, a GP’s mostly just sitting at their computer typing away and asking you to be quiet […]. [At PRONTO!] there was, you know, greater structured time for an actual conversation

– Lachlan, round one, 44 years

Not all participants wished to engage in conversations about sexual health with the peer test facilitators. A few men described the experience of waiting for the test result as awkward and stated they would have preferred to leave the consultation room until the HIV RPOC test result was ready. Nevertheless, one of these participants noted that he still found his conversation at PRONTO! more comfortable than at clinical testing services.

While the HIV RPOC test consultation structure allowed time for conversation, the perceived comfort and value of these conversations largely relied on the quality of the client-provider relationship. Several participants described how, because they felt more comfortable discussing their sexual history with peer at PRONTO!, they had disclosed greater detail about their sexual practices than they had done or would do in clinical services:

it’s not usually about sexual behavior with my GP, in fact it would probably be awkward if he started asking me about things that I’d rather not tell him so I’d much rather just have the blood test and get the hell out of there, whereas I have no problem or I had no problem with the peers talking about what I was up to and why I was there

– Daniel, round one, 39 years

Furthermore, some participants described greater willingness to engage in conversations about sexual health information at PRONTO!. This included feeling comfortable enough at PRONTO! to ask questions they would not have asked at clinical services and giving greater weight to information provided by peer test facilitators:

I liked that you could ask them questions that you probably wouldn’t ask a medical professional […] I could have asked him anything and not felt, not felt stupid

– Neil, round one, 25 years

I just want to hear it anyway just in case, ‘cause I’ve never heard it coming from a peer

– Hugh, round one, 24 years

A few participants across all focus groups described the combination of the consultation structure and the peer relationship as providing an opportunity to discuss other concerns not directly related to HIV and sexual health. Some participants appreciated the opportunity to discuss their emotional or mental wellbeing with test facilitators at PRONTO!, discussions they felt reluctant to have with a clinician. For these participants, the client-provider relationship at PRONTO! provided an opportunity to discuss broader issues, without the need to seek out specialist services such as counselors:

[at a sexual health clinic] if you need to ask questions you can but it’s more about the medical stuff […] and, you know, if you have any other issues they’ll have to send you over there, you know, ‘I’ll have to send you to the counselor’, well, you know, I don’t really want that I just want a chat about something like and I felt like I could just talk, which was good. It was really comfortable

– Evan, round one, 25 years

## Discussion

This is the first study in Australia to examine the acceptability of peer staff in an HIV RPOC testing service for GBM. Findings indicate a high level of acceptability of the peer HIV testing model and broader HIV prevention benefits beyond those conveyed by the provision of a convenient service model for HIV testing. Evaluation survey respondents commonly reported improved HIV risk reduction knowledge, particularly younger and less gay community-attached GBM. The focus group participants identified aspects of the PRONTO! model that encouraged knowledge transfer and open discussions about sex, sexual health and clients’ general wellbeing. These beneficial attributes of a peer-delivered HIV RPOC testing model were juxtaposed against what were perceived as more constraining conventional clinical environments.

Our results suggest that key features of the peer-delivered HIV RPOC testing model were highly acceptable to GBM. While previous research has suggested that conventional sexual health testing is equally acceptable to GBM whether delivered by peers or conventional clinical staff [37], in our study most survey respondents in our study preferred peer-delivered testing. It is encouraging that this preference was largely consistent across participant characteristics. Notably, however, men reporting recent group sex were less likely to report preferring testing with peers. Although not specifically examining group sex, Australian studies indicate that GBM with greater numbers of sexual partners are more likely to have tested for HIV recently [18, 36] and may have more entrenched testing routines [38]. It is possible that men reporting group sex in our study represent a group who may already be comfortable routinely testing at a general practice or sexual health service and for whom the peer model may offer fewer advantages over clinical testing services. Nonetheless, over 80% of participants reporting group sex also reported they would return to PRONTO! in preference to other services, suggesting the peer model does not pose a significant barrier to accessing testing for these men.

A preference for testing with peers among most evaluation survey participants suggests that peer-led HIV RPOC testing may support greater testing frequency. Focus group participants reported finding PRONTO! a more comfortable environment than conventional clinical services for HIV testing because it is a service dedicated to and run by GBM with what was perceived as a more informal, conversational and equal client-provider relationship. Participants contrasted testing at PRONTO! with clinical HIV testing services where they sometimes felt judged when discussing their sexual history, a known barrier to testing for Australian GBM [21, 22]. There is a paucity of literature on the impact of peer-delivered services on HIV testing frequency, however the recent finding that greater proportions of early HIV diagnoses were made among GBM attending an Australian peer-led sexual health service, compared to conventional clinical services, suggests a link between the peer model and HIV testing behavior [28]. There is no evidence yet that PRONTO! has increased testing frequency, and we have previously reported that during the first 12 months of operation only 23% of PRONTO! clients returned to test within six months of their first test [31], compared with 36% returning within six months at local conventional clinical services [19]. In that study the most likely factor identified as affecting return testing was the inconvenience of not offering other STI testing at PRONTO! during the study period, rather than the peer model. However, with the recent introduction of full STI screening, parallel HIV serology and a PrEP clinic at PRONTO!, the impact of the highly acceptable peer model on HIV testing frequency may yet be seen.

By normalizing discussions and improving knowledge about HIV risk and prevention, the peer model at PRONTO! presents a useful opportunity for health promotion. Focus group participants described feeling more comfortable discussing their sexual history in depth and asking relevant questions of peer test facilitators, compared with experiences in clinical HIV testing services. Furthermore, half of the survey participants reported learning something new at PRONTO! about HIV risk or risk reduction. Peer education has a long history as a tool to improve HIV knowledge in diverse groups, including GBM [35, 39, 40]. Our findings suggest that peer-delivered HIV RPOC testing may provide ideal opportunities to engage GBM clients in useful conversations about HIV, grounded in shared client-provider experiences. Our results suggest that these conversations may be particularly useful for younger men and those with fewer gay friends—groups who typically have poorer access to reliable sources of information about HIV prevention and are less likely to have established HIV testing routines [36, 41]. Recent paradigm changes in HIV prevention with the advent of treatment as prevention and PrEP highlight the contemporary importance of having access to reliable sources of up to date information on HIV prevention. In addition, these prevention strategies involve a degree of complexity beyond those needed for traditional primary prevention approaches such as condoms. The capacity for face-to-face and in-depth interactions with GBM provided through a peer HIV RPOC testing model offers a potentially important opportunity to enhance community understanding of new HIV prevention options. At an aggregate level, these discussions may also provide useful insights for policy and program makers into emerging trends and practices within GBM communities to help adapt and refine programs in a time of rapid change [39].

Our study has some limitations. First, the GBM who participated in this study may not represent the broader GBM community. It is possible that GBM who favored the peer model self-selected to test at PRONTO! while those uncomfortable with the peer model may have been less likely to attend. However, only half of the participants in the second evaluation survey reported knowing PRONTO! was a peer-delivered service when they first attended (a question not asked in survey round one) [42]. Second, the PRONTO! clients who participated in surveys and focus groups may not be representative of the broader PRONTO! clientele, with those who engaged more strongly with the service and staff perhaps more likely to participate. Nonetheless, comparison with sentinel surveillance data suggests the survey sample was largely representative of all GBM attending PRONTO! except that greater proportions of survey participants reported a suite of sexual risk behaviors. Third, because the evaluation surveys were not identifiable some clients may have participated in both survey rounds.

## Conclusions

Our study findings demonstrate that peer-led HIV RPOC testing models are highly acceptable to Australian GBM and offer ancillary psychosocial HIV prevention benefits when compared to conventional clinical models of HIV testing. Our findings suggest that peer-delivered HIV RPOC testing can overcome identified barriers to frequent testing among a sizable proportion of GBM who prefer this service model. In addition, our findings suggest that peer-delivered HIV RPOC testing can be a useful opportunity for GBM to openly discuss HIV prevention and their sexual practices in detail, helping develop their HIV risk reduction knowledge. Hence peer models for HIV RPOC testing are likely to be a valuable addition to other local HIV testing services, making a broader contribution to HIV prevention strategies than supporting greater HIV testing uptake alone.
